# The Neural Basis of Social Influence in a Dictator Decision

**DOI:** 10.3389/fpsyg.2017.02134

**Published:** 2017-12-06

**Authors:** Zhenyu Wei, Zhiying Zhao, Yong Zheng

**Affiliations:** ^1^Center for Studies of Education and Psychology of Ethnic Minorities in Southwest China, Southwest University, Chongqing, China; ^2^Key Laboratory for NeuroInformation of Ministry of Education, School of Life Sciences and Technology, University of Electronic Science and Technology of China, Chengdu, China; ^3^Key Laboratory of Cognition and Personality – Ministry of Education, Faculty of Psychology, Southwest University, Chongqing, China

**Keywords:** social influence, equitable decision, norm violation, reward processing, fMRI

## Abstract

Humans tend to reduce inequitable distributions. Previous neuroimaging studies have shown that inequitable decisions are related to brain regions that associated with negative emotion and signaling conflict. In the highly complex human social environment, our opinions and behaviors can be affected by social information. In current study, we used a modified dictator game to investigate the effect of social influence on making an equitable decision. We found that the choices of participants in present task was influenced by the choices of peers. However, participants’ decisions were influenced by equitable rather than inequitable group choices. fMRI results showed that brain regions that related to norm violation and social conflict were related to the inequitable social influence. The neural responses in the dorsomedial prefrontal cortex, rostral cingulate zone, and insula predicted subsequent conforming behavior in individuals. Additionally, psychophysiological interaction analysis revealed that the interconnectivity between the dorsal striatum and insula was elevated in advantageous inequity influence versus no-social influence conditions. We found decreased functional connectivity between the medial prefrontal cortex and insula, supplementary motor area, posterior cingulate gyrus and dorsal anterior cingulate cortex in the disadvantageous inequity influence versus no-social influence conditions. This suggests that a disadvantageous inequity influence may decrease the functional connectivity among brain regions that are related to reward processes. Thus, the neural mechanisms underlying social influence in an equitable decision may be similar to those implicated in social norms and reward processing.

## Introduction

Previous studies have found that humans’ decision-making behaviors are sensitive to inequality considerations, and display a social preference for reducing inequity in distributions (Adams, 1965; Fehr and Schmidt, 1999; Bolton and Ockenfels, 2000; Tricomi et al., 2010). Equity theory indicates that people feel distressed when faced with inequity, and therefore will respond more negatively to inequitable outcomes than to equitable outcomes (Adams, 1965). Using experimental games with real rewards, researchers have also found that people will sacrifice benefits to themselves both when they are offered less than a recipient (disadvantageous inequity) as well as when they are offered more (advantageous inequity) (Loewenstein et al., 1989; Camerer, 2003; Fehr and Fischbacher, 2003; Dawes et al., 2007). One such game, the Dictator Game, has been used to study individuals’ responses to inequity (Fehr and Camerer, 2007). There are two players, the dictator and the recipient, in the original Dictator Game. The dictator need to split an amount of money between herself/himself and the recipient. On average, subjects were found to give 25% of the total to the recipient, which is not a rational decision (Forsythe et al., 1994; Hoffman et al., 1996).

Social influence theory asserts that human beliefs and behaviors can be affected by the preferences and behaviors of others (Cialdini and Goldstein, 2004; Morgan and Laland, 2012; Haun et al., 2013; Toelch and Dolan, 2015). Social conformity is a kind of social influence, and it refers to the action of altering one’s own choice or opinion to align with peers (Turner, 1991). Previous studies have found the effect of social conformity in many domains, including in reconstructing memory, donating to charity, voting, expressing prejudice, investing in the stock market, and pain perception (Sherif, 1936; Asch, 1951; Craig and Prkachin, 1978; Reingen, 1982; Wright et al., 2000; Meade and Roediger, 2002; Walther et al., 2002; Hong et al., 2004; Apfelbaum et al., 2008; Gerber et al., 2008). There are two types of social influence, informational and normative social influence (Deutsch and Gerard, 1955). Informational social influence is defined as an influence to accept information possessed by others as correct behavior, while normative social influence can be defined as an influence to conform to the positive expectations of another (Deutsch and Gerard, 1955). For some intellective decisions, the goal of the decision is to find the correct answer, whereas for a judgment decision, there is no correct answer, and the goal of the decision is to make the “proper” or “preferred” choice (McGrath, 1984; Kaplan and Miller, 1987). Informational influences should predominate when the decision is intellective, whereas normative influences should predominate in judgmental decisions, as these decisions are supported by an appeal to social norms and the consensus preference (Kaplan and Miller, 1987).

Not only the type of social influence, but also the neural mechanisms underlying social influence is of interest. Previous studies have found that participants’ initial judgments could be influenced by social information. Additionally, the choices of group members could affect the neural basis of the low-level processing in music valuation task and mental rotation task (Berns et al., 2005, 2010). The other researcher found that the ventral striatum and the posterior area of medial frontal cortex were activated when an individual experienced conflict with peers’ opinions (Klucharev et al., 2009). A follow-up theta-burst transcranial magnetic stimulation (TMS) study found that transient down-regulation of the posterior medial frontal cortex could reduce conformity behavior (Klucharev et al., 2011). This result suggested that social influence may be supported by a fundamental performance-monitoring neural mechanism (Klucharev et al., 2011). Zaki et al. (2011) found that the nucleus accumbens and the orbitofrontal cortex were involved in social influence effect. These two brain regions were associated with the coding of subjective value.

Wei et al. (2013) designed a modified Ultimatum Game in which participants could observe the decision of peers in the fMRI scanner. They found that participants altered their choices when their decisions conflicted with the collective group behavior in unfair treatment situations. fMRI data indicated that the middle frontal gyrus (MFG), middle temporal gyrus (MTG), insula, inferior parietal lobule (IPL), medial prefrontal cortex (mPFC), and precuneus were activated when participants experienced conflict with group norms. Previous studies have found that these brain areas were associated with behavioral adjustments, reward processing and norm violations (Berns et al., 2001; Holroyd and Coles, 2002).

In the highly complex social environment, our opinions and behaviors can be affected by social information. Although numerous studies have investigated the neural mechanisms underlying social influence by using a variety of judgment tasks, the impact of social influence on equitable decisions remain unknown. In current study, we used a modified dictator game to investigate the impact of social influence on making an equitable decision. The modified dictator game was developed by Zaki and Mitchell (2011). Firstly, we assumed that participants would conform to the choices of group members when these choices are deemed equitable. Secondly, we hypothesized that inequitable group choices would activate the brain regions that are associated with norm violations and reinforcement learning, such as the dorsomedial prefrontal cortex (dmPFC) and the rostral cingulate zone (RCZ). We further reasoned that, if brain regions that are related to norm violations are indeed associated with the effect of social influence on an equitable decision, neural responses in these brain regions would predict individuals’ subsequent choices in decision-making. Finally, there are different mechanisms underlying advantageous inequity and disadvantageous inequity (Fliessbach et al., 2012). Individuals need to deal with incongruences between their sense of fairness and self-interest concerns when they are exposed to an advantageous inequity situation (Loseman et al., 2009). Therefore, we assumed that a psychophysiological interaction (PPI) analysis may confirm increased functional connectivity among brain regions that are related to self-interest and norm violations when subjects are faced with the influence of an advantageous inequity. A previous study has shown that activity in brain regions related to reward processes is reduced in disadvantageous inequity and that this form of inequity can elicit greater dissatisfaction than advantageous inequity (Fliessbach et al., 2012). We therefore hypothesized that a disadvantageous inequity influence would reduce the functional connectivity among brain regions that are involved in reward processes.

## Materials and Methods

### Participants

Twenty-eight healthy right-handed participants (mean age = 22.6, female = 14) completed the experiment. They were native Mandarin speakers, with no neurological illness as confirmed by psychiatric clinical assessment or psychological disorders, and with normal color vision. Data from two participants were excluded from the study. One participant’s head movements exceeded 2.5 mm. The other one subjects misunderstood the rule of experiment. Therefore, there were 26 participants in final analyses (13 males). This study was performed in accordance with the recommendations of the Ethics Committee of Southwest University with written informed consent from all subjects. All subjects gave written informed consent in accordance with the Declaration of Helsinki. The protocol was approved by the Ethics Committee of Southwest University.

### Task Design and Procedure

The present experiment was a two-factor within-participant study, with four levels of peers’ choice (selfish influence, generous influence, intermediate influence, and no-social influence) and two types of offer (selfish offer and generous offer). A selfish influence in a generous offer condition can be defined as an advantageous inequity, while a generous influence in a selfish offer condition is a disadvantageous inequity.

Deception was used in present study (Supplementary Data Sheet 1). Participants were told that they would complete a monetary task with another four group members in the scanner. All of them played the game as a dictator. The four group members would stay in separate behavioral labs. In present study, participant would independently decide how to split a sum of money between herself/himself and a human recipient. The recipient would stay in the fMRI waiting room. All participants in present experiment did not know anything about each other and we told participants that they would not meet each other in the future. About the task, we told participants that they would make iterative distribution decisions about whether to send money to their own accounts or to the partner’s account. After the experiment, computer would choose the results of five of their decisions and added to their final payment. We told participants that the partner would not know what they did in the task, and that the additional payment would simply be transferred into the partner’s account after the experiment. In this way, we can minimize the reputation effects. During the experiment, by using a local network, participant can see peers’ decision. However, peers do not know what participants chose in the task. Additionally, participants would see four “×” symbols instead of peers’ choices if the offer has not been done by all members. We have used this instruction in previous similar studies and it can make participants believe in the existence of peers (Wei et al., 2013, 2016).

**FIGURE 1:**
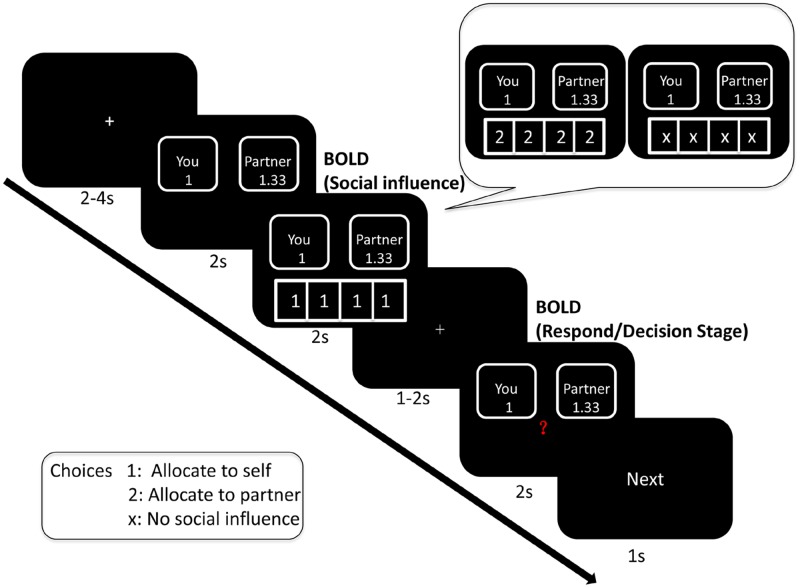
Illustration of sequence of stimuli in one trial (take generous offer trial for example).

Before the experiment, we told participants how the task would proceed. Firstly, participants saw a fixation point for 2–4 s. Then, the offer would be shown on the screen for 1–2 s. Next, they saw peers’ decision underneath the offer for 2 s. Again, a fixation point would be shown on the screen for 1–2 s. In the end, participants responded to the offer when they saw a red question mark in the middle of the screen. After the decision screen, the word “Next” would be shown on the screen for 1 s. This meant that the next offer would be coming shortly. In the scanner, there was an MRI-compatible button box. Participants pressed the button “1” with their right index finger and the button “2” with their middle finger (button “1” refers to self and button “2” refers to the partner). The sequence of events in one trial was illustrated in **Figure 1**.

We used E-Prime 2.0 to present the stimuli and acquire the responses of the participants. In the scanning room, there is a mirror on the top of the image acquisition coil. It can reflect the screen placed at the back of the fMRI scanner. Participants saw the stimuli in the mirror.

### Stimulus Materials

About the peers’ decision, the number “1” indicated a choice to allocate money to self, and the number “2” indicated a choice to allocate money to the receiver. Four conditions of social influence were tested: selfish influence (three or four peers allocated money to themselves); intermediate influence (two peers allocated money to themselves); generous influence (three or four peers allocated money to receiver); and no-social influence condition (the four numbers were replaced with “×”). Intermediate influence trials should be excluded from data analysis.

According to previous studies, the offers were made based on six ratios: 3:1, 2:1, 3:2, 4:3, 5:4, and 1:1, in either person’s favor, for a total of 11 possible ratios (Zaki and Mitchell, 2011; Wei et al., 2016). In each decision, a first value was chosen between ¥0.00 and ¥3.00 at random. Then the second value was determined by the ratio that applied in that trial. For instance, in one trial, the first value ¥2.00 was chosen and the ratio was 2:1, the second value was automatically set as ¥1.00. The amount that either subject or partner stood to gain should be less than ¥9.00 in one trial.

The task had three blocks (40 trials each, i.e., 120 trials in total). One trial lasted 13 s on average. In our study, equity refers to “impartially allocate resources to the person who stood to gain the most” (Zaki and Mitchell, 2011). If the offer adhered to 3:1, 2:1, 3:2, 4:3, or 5:4, it was a selfish offer. It would be equitable if participants allocate money to themselves. If the offer adhered to 1:3, 1:2, 2:3, 3:4, or 4:5, it was a generous offer and it would be equitable if participants allocate money to the receiver. If the offer adhered to 1:1, it was an equal offer. Furthermore, “pure-self” and “pure-other” offers were included in the task (Zaki and Mitchell, 2011; Wei et al., 2016). In the “pure-self” offer trial, a non-zero amount of money was chosen for the participant and ¥0.00 was chosen for the partner (Zaki and Mitchell, 2011; Wei et al., 2016). In the “pure-other” offer condition, ¥0.00 was chosen for the participant and a non-zero amount of money was chosen for the partner (Zaki and Mitchell, 2011; Wei et al., 2016). Moreover, we added zero offer. ¥0.00 was chosen for both the participant and the partner. Consequently, there were 50 selfish offers, 50 generous offers, 10 equal offers, 10 “pure-self” offers, 10 “pure-other” offers, and 10 zero offers in present task (Wei et al., 2016). In the generous offer condition and selfish offer condition, each of them had 15 generous influence trials, 15 selfish influence trials, 15 no-social influence trials, and 5 intermediate influence trials. We only analyzed the selfish offer and generous offers trials.

### Neuroimaging Acquisition and Analysis

Functional MRI data were acquired using a 3T Siemens Trio scanner. Each scan contains 435 functional volumes, using an echo-planar imaging (EPI) sequence with the following parameters: TR/TE = 2000/30 ms, flip angle = 90°, acquisition matrix = 64 × 64, FOV = 192 mm × 192 mm, axial slices = 32, thickness/gap = 3 mm/1 mm, voxel size = 3 mm × 3 mm × 3 mm. The first three images were discarded for the saturation effect.

Image preprocessing was performed with statistical parametric mapping 8 (SPM8; Welcome Department of Imaging Neuroscience, University of London, London, United Kingdom). Functional images were first corrected for motion artifacts. Then images were interpolated to correct for slice timing, and spatially normalized into the Montreal Neurological Institute (MNI)-space using the SPM8 EPI template, and resampled into 3 mm × 3 mm × 3 mm voxels. Images were smoothed using an 8 mm three full-width-at-half-maximum (FWHM) Gaussian kernel.

Statistical analysis was performed in a general linear model in SPM8. The regressors were included based on offers (selfish offers and generous offers), social influence (selfish influence, generous influence, and no-social influence), and a combination of these factors. These regressors were then convolved with the standard hemodynamic response function. In addition, the realignment parameters were included to regress out potential movement artifacts.

For a whole-brain analysis, the results from random effects analyses were all thresholded at *p* < 0.001 (uncorrected). For explore whether the offer type can affect the brain responses to social influence, we analyzed the interaction among the offer type (within subjects factor: selfish, generous) and the social influence (within subjects factor: selfish, generous and no-social influence). Then, for more details insights into which brain regions play a critical role in advantageous inequity influence, we contrasted brain responses to generous offer-selfish influence trials with generous offer-no-social influence. We were also interested in the neural mechanisms underlying disadvantageous inequity influence. Therefore, we contrasted brain responses to selfish offer-generous influence trials with selfish offer-no-social influence. Finally, we analyzed a 2 (offer: selfish, generous) × 3 (social influence: selfish, generous and no-social influence) × 2 (choice: self, receiver) ANOVA in the decision phase.

Psychophysiological interaction analysis was used to assess the connectivity between regions of interest (ROI) and the rest of the brain in response to the experimental condition (Friston et al., 1997). In present study, the ROIs were selected from the brain regions activated in the previous GLM analyses. Dorsal striatum and mPFC have been implicated in reward processes (Euston et al., 2012; Báez-Mendoza and Schultz, 2013). Therefore, based on previous studies and our fMRI results, the dorsal striatum (advantageous inequity influence versus no-social influence; peak at MNI [9, 3, 15]) and mPFC (disadvantageous inequity influence versus no-social influence; peak at MNI [-9, 60, 18]) were selected as seed regions for the PPI analyses. The time series was extracted from each subject in the ROI. And the PPI regressor was then calculated as the element-by-element product of the mean-corrected activity of ROI and a vector coding for differential task effects. The PPI regressors reflected the interaction between psychological variable and the activation time course of the ROI. The individual contrast images reflecting the effects of the PPI between the ROIs and other brain areas were subsequently subjected to one-sample *t*-tests. The results of the group analysis identified brain regions in which the activity systematically showed functional connectivity with the dorsal striatum activity during advantageous inequity influence compared to no-social influence condition, and indicated the functional connectivity between mPFC and other brain regions during disadvantageous inequity influence compared to no-social influence condition.

## Results

1.3% of total trials were excluded from data analyses because participants did not respond in 2 s.

### Behavioral Results

A 3 (social influence: generous, selfish, and no-social influence) × 2 (offer type: generous, selfish) ANOVA was used to analyze the participants’ choices (the rate of allocate money to receiver^1^). We found that the main effect of the factor offer type was significant, *F*(1,25) = 62.37, *p* < 0.001. Participants sent money to partner in generous offer trials (*M* = 0.65, *SD* = 0.05) at a significantly higher rate than the selfish offer trials (*M* = 0.16, *SD* = 0.03). Also, the main effect of the factor social influence was significant, *F*(2,24) = 6.247, *p* < 0.01. Subjects distributed money to partner in generous influence trials (*M* = 0.48, *SD* = 0.04) at a significantly higher rate than the selfish influence trials (*M* = 0.35, *SD* = 0.03) and no-social influence trials (*M* = 0.37, *SD* = 0.04).

**FIGURE 2:**
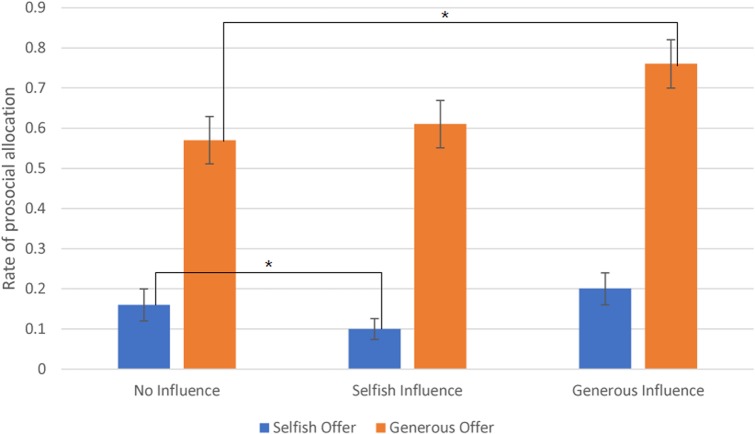
The rate of allocate money to partner (Error bars indicate standard errors of the mean). ^∗^*p* < 0.05.

The interaction effect between offer type and social influence was significant, *F*(2,24) = 9.811, *p* < 0.001. The *post hoc* results showed that subjects sent money to partner in generous offer-no-social influence condition (*M* = 0.57, *SD* = 0.3) at a significantly higher rate than in the selfish offer-no-social influence condition (*M* = 0.16, *SD* = 0.2). Additionally, in generous offer trials, subjects chose to allocate money to receiver at a significantly higher rate in generous influence trials (*M* = 0.76, *SD* = 0.3) than in no-social influence condition. The difference between selfish influence trials (*M* = 0.61, *SD* = 0.3) and no-social influence condition was not significant. In selfish offer trials, participants chose to allocate money to partner at a significantly higher rate in no-social influence trials than in selfish influence trials (*M* = 0.1, *SD* = 0.13). The difference between generous influence trials (*M* = 0.2, *SD* = 0.21) and no-social influence condition was not significant (see **Figure 2**).

### fMRI Results

#### Social Influence Stage

##### Social influence effect

To assess brain regions related to the effect of social influence in the equitable decision, we conducted a 3 (social influence: generous, selfish and no-social influence) × 2 (offer type: generous, selfish) ANOVA. The main effect of offer type was significant in the bilateral cuneus, midbrain, inferior frontal gyrus (IFG), mPFC, IPL, MFG, MTG, and middle occipital gyrus (MOG) (see **Table 1**). The results also indicated that the main effect of social influence was significant in the insula, mPFC, MFG, bilateral MTG, superior frontal gyrus (SFG), IFG, inferior temporal gyrus (ITG), bilateral IPL, cuneus, parahippocampal gyrus, postcentral gyrus, posterior cingulate, and cingulate gyrus (see **Table 2**). Compared with selfish influence and generous influence, cingulate gyrus, IPL and cuneus were active in the no-social influence condition. The activation in mPFC, IFG, SFG, bilateral MFG, and bilateral MTG were more activated in generous influence.

**Table 1:** Significant activation clusters for the main effect of the factor offer type.

Brain regions	HEM	*X*	*y*	*z*	No. of voxels	*t*-value
Cuneus	R	12	-78	6	189	48.34
Midbrain	R	3	-39	-6	106	26.23
Cuneus	L	-6	-81	3	133	35.75
IFG	R	57	12	24	127	24.65
mPFC	L	-3	18	45	442	36.01
IPL	R	45	-45	54	243	22.84
MFG	L	-30	-9	60	250	42.96
MTG	L	-54	-48	-15	19	14.33
MOG	L	-45	-84	9	41	13.58

*Voxels were selected for *p* < 0.05, cluster size > 10, FDR correction. HEM, hemisphere; IFG, inferior frontal gyrus; mPFC, medial prefrontal cortex; IPL, inferior parietal lobule; MFG, middle frontal gyrus; MTG, middle temporal gyrus; MOG, middle occipital gyrus*.

**Table 2:** Significant activation clusters for the main effect of the factor social influence.

Brain regions	HEM	*x*	*y*	*z*	No. of voxels	*t*-value
MTG	R	51	3	-30	45	17.37
MTG	L	-51	6	-30	28	9.89
Insula	L	-33	21	-21	22	15.62
ITG	L	-54	-9	-24	16	10.75
IFG	R	42	24	-12	26	11.98
IFG	L	-54	21	6	63	11.39
Parahippocampal gyrus	L	-27	-45	-18	36	10.86
Parahippocampal gyrus	R	21	-36	-15	19	11.53
Posterior cingulate	L	-6	-48	3	60	10.41
MTG	R	63	-33	-12	29	10.56
MTG	L	-54	-69	30	12	9.14
SFG	L	-18	33	21	16	8.78
Cuneus	R	3	-81	36	117	14.27
IPL	R	57	-36	36	68	13.39
IPL	L	-69	-33	30	46	8.26
mPFC	R	6	51	42	248	15.82
MFG	L	-39	18	42	137	12.19
MFG	R	30	42	33	13	8.36
MFG	R	45	45	-15	31	13.28
Cingulate gyrus	R	6	-24	45	65	13.65
Postcentral gyrus	R	57	-15	51	10	7.43

*Voxels were selected for *p* < 0.05, cluster size > 10, FDR correction. HEM, hemisphere; MTG, middle temporal gyrus; IFG, inferior frontal gyrus; ITG, inferior temporal gyrus; SFG, superior frontal gyrus; MFG, middle frontal gyrus; IPL, inferior parietal lobule; mPFC, medial prefrontal cortex*.

**FIGURE 3:**
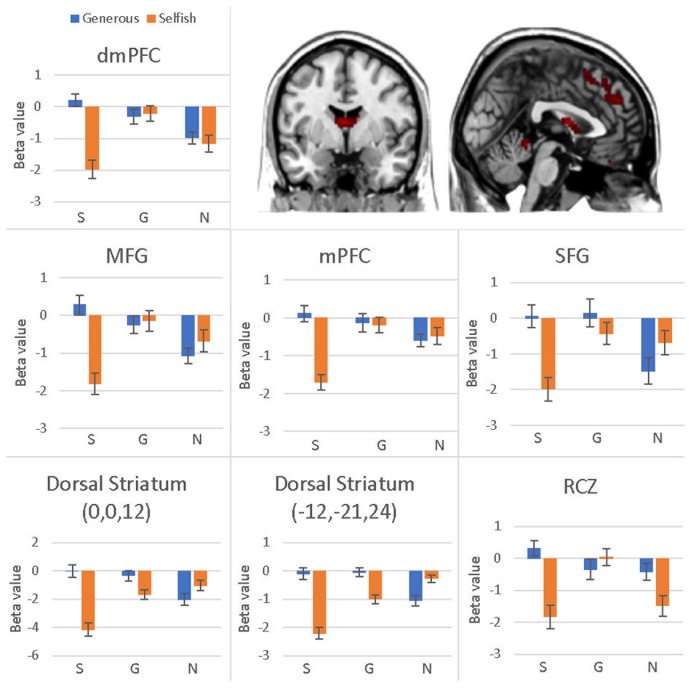
Brain regions involved in the interaction effect between offer type and social influence, *p* < 0.05, cluster size = 10, FDR correction. *Y*-axis represents the beta value. G, generous influence; S, selfish influence; N, no social influence. (Error bars indicate standard errors of the mean.)

The interaction effect was significant in the mPFC, bilateral caudate, dmPFC, RCZ, MFG, SFG, cingulate gyrus, IPL, and bilateral postcentral gyrus (see **Table 3** and **Figure 3**). *Post hoc* contrast indicated that mPFC, dmPFC, RCZ, MFG, and SFG were activated when the offer is generous and the social influence is selfish. The bilateral caudate were deactivated when the offer is selfish and the social influence is selfish. This result indicated that subjects might simply follow their group members therefore they offloaded the computation of value of decision choices from their brain (Engelmann et al., 2009).

**Table 3:** Significant activation clusters for the interaction between offer type and social influence.

Brain regions	HEM	*x*	*y*	*z*	No. of voxels	*t*-value
mPFC	R	33	57	-3	20	11.69
dmPFC	R	3	48	33	73	12.32
RCZ	R	0	15	57	7	9.49
Dorsal striatum	R/L	0	0	12	53	15.23
Dorsal striatum	L	-12	-21	24	9	17.32
MFG	R	42	27	36	130	13.01
MFG	L	-36	33	36	6	8.78
SFG	L	-27	54	-3	14	9.27
Cingulate gyrus	R	21	-33	24	9	15.46
Postcentral gyrus	L	-15	-33	72	26	13.08
Postcentral gyrus	R	27	-51	72	5	10.02
IPL	L	-57	-60	39	7	9.66

*Voxels were selected for *p* < 0.05, cluster size > 10, FDR correction. HEM, hemisphere; mPFC, medial prefrontal cortex; dmPFC, dorsomedial prefrontal cortex; RCZ, rostral cingulate zone; MFG, middle frontal gyrus; SFG, superior frontal gyrus; IPL, inferior parietal lobule*.

##### Advantageous inequity influence effect

**FIGURE 4:**
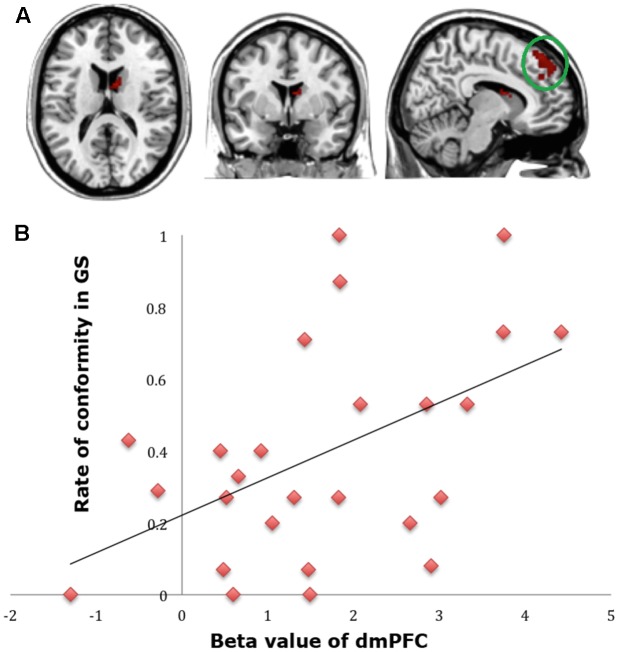
**(A)** Brain regions involved in advantageous inequity influence (generous offer–selfish influence > generous offer–no social influence), *p* < 0.001, cluster size = 10, uncorrected. dmPFC was marked with a green circle. **(B)** dmPFC β-values for the advantageous inequity influence effects were positively correlated with the rate of allocate money to self when offer is generous and social influence is selfish.

**FIGURE 5:**
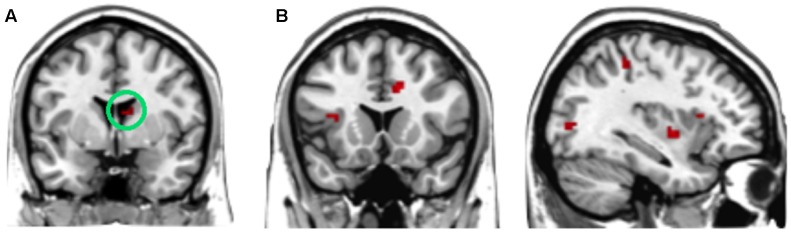
**(A)** The region of interest was marked with a green circle (dorsal striatum, peak at MNI coordinates: [9, 3, 15]). **(B)** Results of psychophysiological interaction (PPI) analysis. Functional connectivity with the dorsal striatum in the advantageous inequity influence effects influence (generous offer–selfish influence > generous offer–no social influence). A threshold of *p* < 0.001, cluster size = 10, uncorrected was used for the conjunction.

To find out which brain regions were associated with the effect of advantageous inequity influence, we compared selfish influence trials with no-social influence trials in the generous offer condition (generous offer-selfish influence > generous offer-no-social influence). As expected, advantageous inequity influence trials induced activation in the dmPFC, caudate, MFG and IPL (see **Table 4** and **Figure 4A**). In addition, we found that the neural response in the dmPFC predicted subsequent conformity behavior when the offer is generous and the influence is selfish. **Figure 4B** illustrates the significant positive correlation between the neural response in the dmPFC and the rate of conformity behavior when the offer is generous and the influence is selfish (*r* = 0.49, *n* = 26, *p* = 0.01). PPI analysis showed that activity in the dorsal striatum was accompanied by task-dependent (advantageous inequity influence > no-social influence) functional interaction with brain areas: bilateral insula, supplementary motor area (SMA), MOG, cingulate gyrus, postcentral gyrus, IPL and precuneus (see **Table 5** and **Figure 5** for more details).

**Table 4:** Significant activation clusters for advantageous inequity influence effect.

Brain regions	HEM	*x*	*y*	*z*	No. of voxels	*t*-value
Dorsal striatum	R	9	3	15	12	4.83
dmPFC	R	3	42	36	96	5.9
MFG	R	45	27	45	49	4.79
IPL	L	-57	-60	39	10	4.61

*Voxels were selected for *p* < 0.001, cluster size > 10, uncorrected. HEM, hemisphere; dmPFC, dorsomedial prefrontal cortex; MFG, middle frontal gyrus; IPL, inferior parietal lobule*.

**Table 5:** Results of PPI analysis of advantageous inequity influence effect.

Brain regions	HEM	*x*	*y*	*z*	No. of voxels	*t*-value
Insula	R	42	9	9	13	4.09
Insula	L	-36	-3	0	7	4.68
Insula	L	-36	18	12	8	4.25
SMA	L	-9	-9	63	19	4.41
Cingulate gyrus	R	12	18	33	19	4.72
IPL	L	-36	-39	51	4	4.05
MOG	L	-39	-81	6	21	4.77
Precuneus	R	24	-60	54	7	4.84
Postcentral Gyrus	L	-45	-30	45	10	4.32

*Voxels were selected for *p* < 0.001, cluster size > 10, uncorrected. HEM, hemisphere; SMA, supplementary motor area; IPL, inferior parietal lobule; MOG, middle occipital gyrus*.

##### Disadvantageous inequity influence effect

**FIGURE 6:**
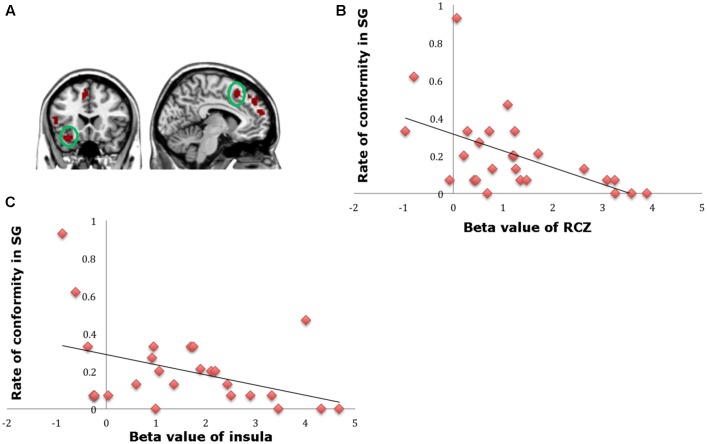
**(A)** Brain regions involved in disadvantageous inequity influence (selfish offer–generous influence > selfish offer–no social influence), *p* < 0.001, cluster size = 10, uncorrected. RCZ and insula were marked with green circles. **(B)** RCZ β-values for the disadvantageous inequity influence effects were negatively correlated with the rate of allocate money to the partner when the offer is selfish and social influence is generous. **(C)** Insula β-values for the disadvantageous inequity influence effects were negatively correlated with the rate of allocate money to the partner when the offer is selfish and social influence is generous.

**FIGURE 7:**
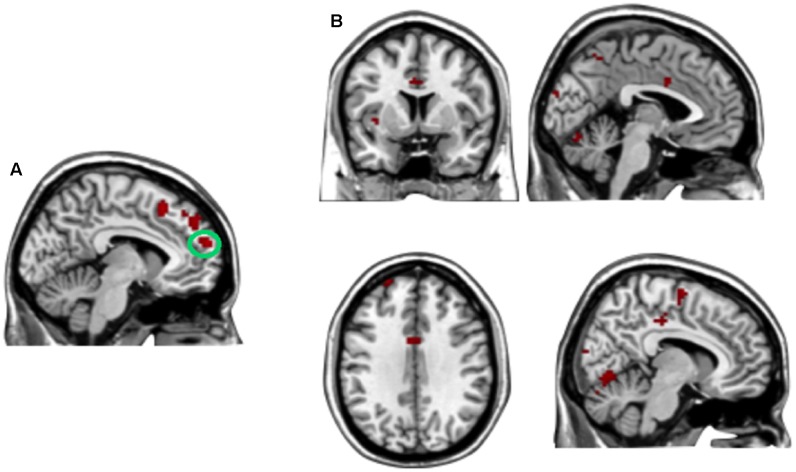
**(A)** The region of interest was marked with a green circle (medial prefrontal cortex, peak at MNI coordinates: [–9, 60, 18]). **(B)** Results of psychophysiological interaction (PPI) analysis. Functional connectivity with the medial prefrontal cortex in the disadvantageous inequity influence effects (selfish offer–generous influence > selfish offer–no social influence). A threshold of *p* < 0.001, cluster size = 10, uncorrected was used for the conjunction.

A direct contrast of generous influence trials with no-social influence trials in the selfish offer condition (selfish offer-generous influence > selfish offer-no-social influence) showed significant activation of the bilateral mPFC, dmPFC, RCZ, insula, bilateral MTG, MFG, MOG, and IFG (see **Table 6** and **Figure 6A**). We also found that the activation in the insula and RCZ predicted individuals’ conformity when the offer is selfish and the influence is generous. **Figure 6B** shows a negative correlation between the neural response in the insula and the rate of conformity when the offer is selfish and the influence is generous (*r* = -0.4, *n* = 26, *p* = 0.04). The other negative correlation was found between the neural response in the RCZ and the rate of conformity when the offer is selfish and the influence is generous (*r* = -0.54, *n* = 26, *p* = 0.004) (**Figure 6C**). By using PPI analysis (disadvantageous inequity influence > no-social influence), we found decreased functional connectivity between mPFC and insula, SMA, SFG, MFG, sub-gyral, cingulate gyrus, dorsal anterior cingulate cortex (dACC), posterior cingulate gyrus (PCG), ITG, MOG, postcentral gyrus, cuneus, precuneus, and bilateral lingual gyrus (see **Table 7** and **Figure 7** for more details).

**Table 6:** Significant activation clusters for disadvantageous inequity influence effect.

Brain regions	HEM	*x*	*y*	*z*	No. of voxels	*t*-value
MTG	R	54	9	-33	19	4.94
MTG	L	-51	-33	-3	19	5.11
MFG	L	-48	42	-15	7	5.35
MOG	R	45	-84	6	11	5.36
IFG	L	-54	21	12	12	4.02
Insula	L	-33	21	-21	6	5.23
RCZ	L	-6	21	51	12	4.81
mPFC	R	9	60	21	8	4.17
mPFC	L	-9	60	18	15	5.47
dmPFC	L	-9	48	39	20	4.48

*Voxels were selected for *p* < 0.001, cluster size > 10, uncorrected. HEM, hemisphere; MTG, middle temporal gyrus; MFG, middle frontal gyrus; MOG, middle occipital gyrus; IFG, inferior frontal gyrus; RCZ, rostral cingulate zone; dmPFC, dorsomedial prefrontal cortex; mPFC, medial prefrontal cortex*.

**Table 7:** Results of PPI analysis of disadvantageous inequity influence effect.

Brain regions	HEM	*x*	*y*	*z*	No. of voxels	*t*-value
Insula	L	-36	9	-3	13	-4.25
SMA	R	15	-6	69	29	-5.8
SFG	L	-24	57	33	10	-4.81
MFG	L	-27	-3	45	12	-4.62
Sub-gyral	L	-18	-12	51	7	-4.96
Cingulate gyrus	R	9	-24	42	10	-4.15
dACC	L	-3	3	33	12	-4.24
PCC	L	-9	-57	3	9	-4.04
ITG	L	-48	-66	-6	7	-3.7
MOG	R	30	-81	6	64	-5.62
MOG	L	-9	-84	15	59	-4.67
Postcentral gyrus	R	66	-12	18	9	-4.38
Cuneus	L	-12	-102	6	15	-4.14
Precuneus	L	-6	-54	54	13	-3.74
Lingual gyrus	L	-18	-93	-18	11	-4.06
Lingual gyrus	R	9	-69	-6	27	-4.64
Lingual gyrus	R	18	-81	-3	19	-4.95

*Voxels were selected for *p* < 0.001, cluster size > 10, uncorrected. HEM, hemisphere; SMA, supplementary motor area; SFG, superior frontal gyrus; MFG, middle frontal gyrus; dACC, dorsal anterior cingulate cortex; PCC, posterior cingulate cortex; ITG, inferior temporal gyrus; MOG, middle occipital gyrus*.

#### Decision Stage

**FIGURE 8:**
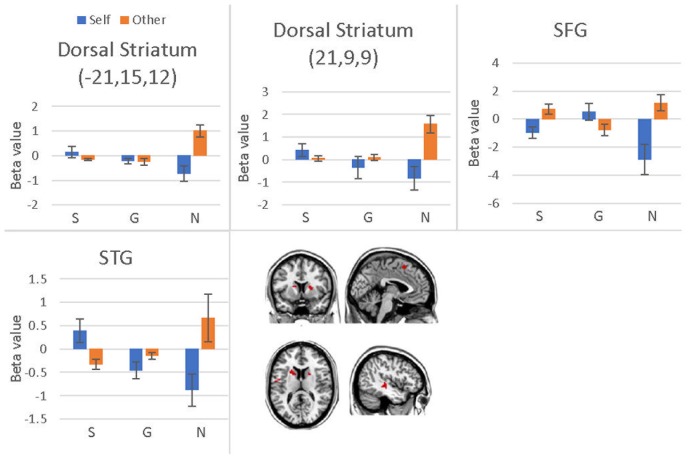
Brain regions involved in the social influence and subjects’ choices interaction, *p* < 0.001, cluster size = 10, uncorrected. *Y*-axis represents the beta value. G, generous influence; S, selfish influence; N, no social influence (Error bars indicate standard errors of the mean).

We conducted a 2 (offer type: generous, selfish) × 2 (choices: self, receiver) × 3 (social influence: generous, selfish, no-social influence) ANOVA. Only the interaction effect between social influence and choices was significant in bilateral dorsal striatum, superior temporal gyrus (STG), SFG, precentral gyrus and postcentral gyrus (see **Table 8** and **Figure 8**). *Post hoc* contrast indicated that the bilateral dorsal striatum were more activated when participants allocated money to receiver than to themselves in the no-social influence trials. In the selfish influence trials, STG was more activated when subjects allocated money to themselves than to receiver. However, in the no-social influence condition, STG was more activated when subjects allocated money to receiver than to themselves. The activity of SFG was significantly more strongly affected by the generous choice than the selfish choice in the selfish influence, as well as in the no-social influence trials. However, it was significantly more strongly affected by the selfish choice than the generous choice in the generous influence condition.

**Table 8:** Significant activation clusters for the three-way ANOVA in decision stage.

Brain regions	HEM	*x*	*y*	*z*	No. of voxels	*t*-value
Dorsal striatum	R	21	9	9	20	9.17
Dorsal striatum	L	-21	15	12	8	11.62
STG	L	-45	-27	0	6	8.61
SFG	L	0	18	54	12	8.74
Precentral gyrus	L	-48	-18	36	12	8.09
Precentral Gyrus	L	-48	-3	15	7	8.12
Postcentral Gyrus	L	-9	-33	66	24	9

*Voxels were selected for *p* < 0.001, cluster size > 10, uncorrected. HEM, hemisphere; STG, superior temporal gyrus; SFG, superior frontal gyrus*.

## Discussion

Our study set out to investigate the effect of social influence on an equitable decision. As humans, our decisions and judgments can be affected by the normative group behavior (Cialdini and Goldstein, 2004; Klucharev et al., 2009). We found that the choices of participants were influenced by the choices of peers in equitable decision. However, participants’ decisions were influenced by equitable rather than inequitable group choices.

Using fMRI, we found out the brain regions that were associated with the social influence on equitable decisions. We found that the group’s inequitable choices activated the dmPFC, RCZ, mPFC, bilateral caudate, bilateral MFG, SFG, cingulate gyrus, bilateral postcentral gyrus, and IPL in the selfish offer condition. In our study, selfish–inequitable influence was defined as disadvantageous inequity, while generous–inequitable influence was defined as an advantageous inequity. A previous questionnaire-based study has shown that participants responded more negatively to disadvantageous inequity than to advantageous inequity (Loewenstein et al., 1989). Disadvantageous inequity conflicts with individuals’ sense of equity and self-interest concern, while advantageous inequity only conflicts with individuals’ sense of equity. In advantageous inequity situations, the equity norm and self-interest are in conflict. Therefore, the evaluation of advantageous inequity requires more cognitive resources than that of disadvantageous inequity (van den Bos et al., 2006; Fliessbach et al., 2012). Researchers suggested that individuals’ reactions to advantageous inequity and disadvantageous inequity are different extremely (Fliessbach et al., 2012).

Previous fMRI studies have found that the RCZ is activated when individuals need to adjust their behaviors (Kerns et al., 2004; Ridderinkhof et al., 2004; Cohen and Ranganath, 2007). It has been shown that the activation of the RCZ is related to the individual’s perception of incongruence in terms of judgments related to unfair distribution (Sanfey et al., 2003), social exclusion (Eisenberger et al., 2003), and social descriptive norms (Klucharev et al., 2009). mPFC and MFG also are involved in detecting norm violations (Berthoz et al., 2002; Falk et al., 2010; Wei et al., 2013). Previous norm violation studies found that a special neural mechanism may exist in the human brain, it can detect norm violation (Montague and Lohrenz, 2007). A study by Beer et al. (2003) supported this hypothesis; they found that patients with damaged mPFC were insensitive to group rule, which implicated the mPFC in norm violation. Another fMRI study also found that the mPFC was involved in normative social influence (Mason et al., 2009). Additionally, activation of the dmPFC has been associated with changes in preference and with cognitive imbalance (Izuma and Adolphs, 2013).

Previous studies have shown that two conflictive motives could affect people’s responses to advantageous inequity: one is the pleasantness of getting a relatively better outcome; the other is the fairness concern (Peters et al., 2004; Loseman et al., 2009). We found that that an advantageous inequity influence was related to activation of the dmPFC, dorsal striatum, MFG, and IPL. These brain areas are known to be associated with changes in preference and in processing conflicting information (Berns et al., 2005; Falk et al., 2010; Izuma and Adolphs, 2013). PPI analysis suggested positive functional connectivity between BOLD activities in the dorsal striatum and those in brain regions related to norm violation (bilateral insula, SMA), among other brain areas. Most importantly, the dmPFC is related to self-oriented behavior and to maximizing one’s own gains (Burnett et al., 2009; van den Bos et al., 2011). Our brain–behavior correlation analysis indicated that activation of the dmPFC significantly predicted the frequency of later conformity in the generous offer–selfish influence condition. The higher the dmPFC activation while viewing others’ selfish–inequitable choices, the more likely individuals were to choose to conform to others’ choices (allocate money to themselves).

Previous studies have suggested that there are two sources of negative emotion can be evoked by disadvantageous inequity: one is the unfair resource distribution; the other is the dissatisfaction for not receiving the good outcome while someone else does (van den Bos et al., 1997; Loseman et al., 2009). In our study, fMRI results suggested that several brain regions, such as the RCZ, dmPFC, insula, bilateral mPFC, bilateral MTG, MFG, MOG, and IFG were involved in the disadvantageous inequity influence. PPI analyses revealed that a negative functional connectivity between the mPFC and insula, SMA, dACC was involved in the disadvantageous inequity influence. These regions have been shown to encode expected reward values, as well as the reward value of outcomes (Bush et al., 2002; Naqvi and Bechara, 2009; Krebs et al., 2011; Rushworth et al., 2011). The PPI results may indicate that the disadvantageous inequity influence may decrease functional connectivity between brain regions that are related to reward processes. Additionally, as we expected, participants who more strongly engaged the insula and RCZ when viewing others’ generous–inequitable choices made fewer conformity choices (allocated money to the receiver) in the selfish offer–generous influence condition. These brain regions are related to error detection (Ridderinkhof et al., 2004; Diedrichsen et al., 2005) and the encoding of inequity (Sanfey et al., 2003; Singer et al., 2006; Hsu et al., 2008; Zaki and Mitchell, 2011; Güroğlu et al., 2014; Yu et al., 2014). The insula is also responsive to disgusting stimuli (Calder et al., 2001) and the RCZ is associated with negative feedback (Ullsperger and Von Cramon, 2004). In the present study, we concluded that, when the offer is a selfish offer, allocating money to the receiver would produce a sense of subjective disutility (Zaki and Mitchell, 2011). The disadvantageous inequity influence may evoke individuals’ aversive emotional states. Participants who demonstrated the strongest negative emotional response to disadvantageous inequity influences were less likely to act generously in the selfish offer condition.

In the decision stage, the fMRI results indicated that the bilateral dorsal striatum, STG, and SFG were activated when individuals made prosocial choices (allocated money to the receiver) in the no-social influence trials, irrespective of the types of offer. In line with the previous studies focused on prosocial decisions, our results indicated that this process might be guided by the ability to shift attention from oneself to the needs and values of other in which mentalizing plays a crucial role. Evidence from neuroimaging study has shown that STG is one of the key brain regions underlying mentalizing and attention shift to focus on the needs of others (Völlm et al., 2006). During prosocial decision making, the striatum has been speculated to represent both monetary and social rewards and associated with the rewards from help others (Moll et al., 2006; Izuma et al., 2010).

A number of limitations in present study should be mentioned. Firstly, the fMRI results of advantageous inequity influence and disadvantageous inequity influence were uncorrected. Secondly, there was only a 2 s inter-stimulus interval between social influence screen and decision screen. A longer ISI could help better to avoid the carry over effect from the social influence screen on the decision screen.

## Conclusion

The present study assessed the neural mechanisms underlying social influence. The results extend our knowledge of equitable decision-making. Equity is said to be a fundamental human need (Camerer, 2003; Fehr and Fischbacher, 2003; Brosnan, 2006). Our behavioral results suggested that people are more likely to conform to peers’ choices if those choices are equitable. The neuroimaging results indicated that brain regions related to norm violations and behavioral adjustment were activated when participants experience an inequitable group opinion. Individuals’ conformity behavior can be predicted by the neural responses in dmPFC, RCZ, and insula. Advantageous inequity is related to significantly increased functional connectivity between the dorsal striatum and brain areas that are associated with norm violation. In contrast, responses to disadvantageous inequity were supported by negative connections between the mPFC and brain regions that are involved in expected reward values. The present results may reflect that the neural mechanisms underlying social influence on equitable decisions may be similar to those previously implicated in social norms and reward processing.

## Author Contributions

Conceived and designed the experiments: ZW and YZ. Program the task: ZW and ZZ. Performed the experiments: ZW. Analyzed the data: ZW and ZZ. Wrote the paper: ZW, ZZ, and YZ.

## Conflict of Interest Statement

The authors declare that the research was conducted in the absence of any commercial or financial relationships that could be construed as a potential conflict of interest.

## Footnotes

1 For each participant, the rate of money allocation to partner was calculated as number of the trials in which participant allocated money to partner divided by the total trial number in each condition.
