# Supplementary material for: The Neural Basis of Social Influence in a Dictator Decision
Source: Front Psychol. 2017 Dec 6;8:2134. doi: 10.3389/fpsyg.2017.02134 (PMC5770631; doi:10.3389/fpsyg.2017.02134)
Supplement: Supplementary file 1 [file Data_Sheet_1.pdf]

Appendix

Welcome to our experiment! You are now in the group 12. There are five participants in this group. All of you will play the game as an allocator. You would independently play a monetary task with your partner, who would stay in the MRI waiting room. In this game, you will make decisions about whether to distribute an amount of money to yourself, or a different amount to your partner. These offers are made by computer.

You will see ‘you’ and ’partner’ on the screen. Above the word ‘you’, there will be an amount of money displayed. Above the word ‘partner’, there will be a different amount of money displayed. These are the amounts you can choose to award to yourself or the partner.

For example, you see ‘1’ above ‘you’, and ‘2’ above ‘partner’. This means that you have the choice as to whether a ¥1 prize will be given to you or a ¥2 prize will be allocated to the partner. You can make these choices by pressing the buttons. If you press button 1, you will award the prize to yourself. If you press button 2, you will give the prize to the partner. 5 trials will be randomly selected and added to your payment.

At the start of each trial, you will see a fixation point. Then, the offer would be shown on the screen. After that, you will see the choices of group members above the offer, followed by a fixation point. In the end, you will see a red question mark on the screen. This means that you need to decide within 2 seconds. The decision screen will be followed by the word ‘Next’.

Remember that:

Your partner will stay in the MRI waiting room. You would have no further interactions with the partner. Your partner does not know you and won’t know your decisions. (S)he will get a bonus in addition to her/his payment after she/he finished a simple task.

Your group members will stay in the separate room. You will see the choices of your group member during the experiment. The order of the trials is random, so sometimes you cannot see their choices if the offer has not been done by your group members. You will see four white crosses in this situation. Your group members would not know your choices.
